# State-of-the-Art Plant Virus Research in Australasia

**DOI:** 10.3390/v15061311

**Published:** 2023-06-01

**Authors:** Steve Wylie, Nuredin Habili

**Affiliations:** 1 The Harry Butler Institute, Murdoch University, 90 South Street, Perth, WA 6150, Australia; 2 The Australian Wine Research Institute, Wine Innovation Central Building, Hartley Grove, Urrbrae, SA 5064, Australia; 3 School of Agriculture, Food and Wine, University of Adelaide, Waite Precinct, PMB 1, Glen Osmond, SA 5064, Australia

The Special Issue ‘State-of-the-Art Plant Virus Research in Australasia’ in *Viruses* provided a fascinating snapshot of plant and fungus virus research being undertaken in Australasia during the final year of the official COVID-19 pandemic. Eight papers published from April 2022 to May 2023 were presented in this Special Issue, all originating from Australia and New Zealand.

Obliquely associated with plant virus research, the first paper in the series [1] provided detailed historical and scientific evidence for the origin of a plant used in most, if not all, plant virology laboratories around the world—*Nicotiana benthamiana*. This variable species has an uneven distribution across Northern Australia (colloquially known as The Outback), but previous sleuthing had indicated the accession used by virologists was unusual in several aspects and originated from a single source at a gold-mining site called The Granites, a remote group of low granite hills located hundreds of kilometres north-west of the nearest large town of Alice Springs. The authors obtained a herbarium specimen of *N. benthamiana*, collected by Dr John Cleland on a trip he undertook to The Granites in 1936 to study a group of Warlpiri Aboriginal people living there, who still maintained a largely traditional hunter–gatherer lifestyle. The Warlpiri used *N. benthamiana* as a source of the addictive alkaloid nicotine, yet maintained taboos against young women using it as such. Nowadays, we all know of the damage to the unborn foetus that tobacco use causes, and it seems that back then, the Warlpiri were well aware of the danger too. The 86-year-old herbarium specimen held the same mutation in its *RdRp1* gene seen in the laboratory accession of *N. benthamiana* today, confirming it to be a ‘natural’ mutation. A paper trail of historical records, scientific publications, and new scientific data led from the *N. benthamiana* population at The Granites in 1936 to Professor Thomas H. Goodspeed at UC Berkeley in 1939, and from there to the Division of Tobacco Investigations in Maryland by 1945, and onwards to its ubiquitous distribution in virology labs today.

The second paper in this series [2] concerned an examination of two subgroups of the cytorhabdovirus lettuce necrotic yellows virus (LNYV), a virus apparently endemic to the Australia/New Zealand region. The original host of the virus is unknown but is presumably a species or group of plant species indigenous to the region. LNYV has spilled over from its original host(s) to damage valuable lettuce crops and the sowthistle weed. The two LNYV subgroups, designated SI and SII, are differentiated by the sequence diversity of the nucleocapsid gene. Of interest is the relative success of SII in Australia compared to SI. Although isolates of both subgroups existed there, now, only SII isolates are found, suggestive of rapid selection for a trait(s) present in SII. In New Zealand, isolates of both subgroups still exist. The authors hypothesise the viral glycoprotein (G) involved in transmission by three non-indigenous aphid vectors is involved in SII’s apparent dominance. The predicted 3-D structures of G-protein domains suggest a role in membrane fusion, possibly allowing entry into vector aphid cells. Two amino acid positions in domain 3 of the SII G protein, and the presence of a disulphide bond providing greater stability, are hypothesised to explain its relative success over SI isolates in Australia. In New Zealand, the authors hypothesise that lower ambient temperatures promote stability in SI isolates, even while lacking the disulphide bond, explaining the presence of both subgroups. This paper provides a fascinating insight into the rapid evolution of an indigenous virus that is emerging into a new suite of exotic vectors and hosts provided by human activities.

The third paper [3] also described the spillover of a virus of the indigenous flora to exotic hosts introduced by humans. *Anthocercis* is a genus of solanaceous shrubs and small trees indigenous to Australia. Yellow tailflower mild mottle virus (YTMMV) is a tobamovirus described from several *Anthocercis* species. The authors examined its response to the presence of the introduced weed *Solanum nigrum*, which sometimes grows in physical contact with infected *Anthocercis* plants. Would YTMMV spillover to *S. nigrum*? Populations of *S. nigrum* growing adjacent to and distant from infected *Anthocercis* plants were examined and found to be infected with YTMMV. The existence of *S. nigrum* populations growing in suburban locations far from *Anthocercis* populations suggested seed-borne transmission and spread of YTMMV facilitated by fruit-eating birds. Seed transmission of YTMMV in *S. nigrum* was then proven in the laboratory. The threat of infection of YTMMV to solanaceous crops such as potato is discussed in light of the export of such crops providing potential opportunities for international travel and spread by YTMMV.

Poleroviruses of peas and other crops and surrounding weeds in Tasmania were the main focus of the fourth paper in the series [4], although other virus groups were also identified. The authors found that turnip yellows virus (TuYV) was the most prevalent polerovirus, infecting 18 of 22 pea fields tested, with an incidence of up to 27% of plants tested at one site. The authors also found the polerovirus brassica yellows virus (BrYV), an isolate of which may be a recombinant with TuYV. Analysis of the polerovirus P0 and P3 genes revealed phylogenetic incongruence, indicating that these genes have distinct evolutionary histories. Additionally, two potyviruses were detected, as well as one species each of a luteovirus, carlavirus, and potexvirus, and an undescribed partitivirus. In contrast to papers 2 and 3, where indigenous viruses were adapting to new hosts, in this paper, exotic viruses were shown to be evolving in exotic hosts under selection pressures present in their new geographical location of Tasmania.

Paper five in the series [5] was about preventing exotic viruses from gaining new geographical territory. The paper presented a bioinformatic workflow for detecting viruses for which no current tests exist. High-throughput sequencing (HTS) is the tool of choice for identifying unknown viruses infecting hosts, but a downside of this approach is that most of the sequenced nucleic acids are of the host, not of the virus of interest. How can viral sequences be quickly and reliably distinguished from host sequences in an automated manner? Many, if not all, of the current virus detection pipelines are inadequate for this task. The authors generated their sequence read datasets using MinION and HiSeq platforms and tested 60 combinations of datasets and bioinformatics tools, some developed in-house, to identify those that were able to reliably identify the viral ‘needle’ in the host ‘haystack’. The authors claim their work provides the basis of an automated analysis pipeline for HTS-based virus diagnostics in a biosecurity setting.

The authors of paper six [6] examined different approaches to preventing new virus incursions of viruses in New Zealand. They, too, recognised the value of HTS to identify new and unexpected viruses and viroids at biosecurity checkpoints, but pointed out the current disadvantages of HTS in terms of cost and time and other resources required. In many cases, rapid on-the-spot diagnosis capability is more appropriate, and HTS cannot be used in such situations. Point-of-use (POU) diagnostic tools that are rapid, cheap and simple to use in situ were discussed. The authors identified recombinase polymerase amplification (RPA) and loop-mediated amplification (LAMP), neither of which require expensive equipment, as fast and very sensitive. They proposed that POU tools such as RPA and LAMP are suitable for wider utilisation in the context of national borders to improve plant virus biosecurity.

Paper seven [7] reported research into mycoviruses (viruses that infect fungi), not plant viruses as such, although in this case, the virus-infected fungus originated from inside the root of an Australian indigenous orchid. The research project was part of an ongoing study into the complex symbiotic relationships between plants, fungi and viruses. Many mycovirus/host relationships are described as ‘latent’, meaning there is no or little impact of infection. In this study, the authors developed isogenic fungal lines either infected with three endornaviruses or uninfected by them. They compared small RNA profiles of each to assess how virus infection influenced gene expression in the host. Of over 3000 host-derived small RNA species identified, about 300 were up-regulated in response to infection, and about 30 were down-regulated. The origins of these RNAs were genes responsible for transcriptional regulation, catalysis, molecular binding, and metabolic activities, emphasising that virus infection of a host is a dynamic interaction and far from ‘latent’.

The final paper [8] in the series was a summary of research carried out over 20 years at Waite Diagnostics, The University of Adelaide, South Australia, on grapevine leafroll-associated virus 2 (GLRaV-2), the only member of genus *Closterovirus* known to infect grapevine. Since 2001, 11,257 vine samples were received, of which 313 tested positive for GLRaV-2, an overall incidence of 2.7%. This incidence rate remained steady over 20 years because this virus does not have a natural vector and spreads mainly through grafts from infected vines. GLRaV-2 isolates exist as six phylogenetic groups based on coat protein and p24 nucleotide sequences, which to some extent reflect their pathogenic properties. The groups are designated PN, 93/955, H4, BD, RG and PT20. Members of PN, which are more prevalent, as well as the 93/955 group, are associated with both leafroll symptoms and graft incompatibility. The BD group appears to be asymptomatic and is rarely associated with leafroll symptoms or with graft incompatibility. The RG group is associated with the graft incompatibility symptoms which only develop following grafting, but it is symptomless in ungrafted vines. Although three different phylogroups of GLRaV-2 were detected in the same vine, no evidence of recombination was observed among them. GLRaV-2 is a debilitating virus, especially in phylloxera-infested regions where the grafting practice is mandatory. The authors propose that in such areas the use of rootstock genotypes sensitive to the virus should be avoided.

In summary, there is investment in a broad range of plant virus-related topics in Australasia. From a biosecurity viewpoint, the region has the advantage of a continuous oceanic border around the island continent of Australia and the islands of New Zealand, which forms a barrier to the natural spread of viruses. However, as the international trade in fresh produce and propagules increases, and the climate continues to change, opportunities for viruses (and other pathogens) to jump over oceanic barriers and expand their ranges to Australasia and its crops and flora will probably increase. Similarly, we have a responsibility to keep the viruses that evolved in the region from escaping and establishing in new continents.

The indigenous viromes of Australasian floras have largely been ignored to date. This is understandable given the limited sources of funding for research in ‘non-commercial’ plants and fungi. However, we urge that a more thorough knowledge of Australasian viromes be obtained in years to come, not only to forestall future spillover events but also simply to pursue knowledge for the sake of knowledge, a noble scientific endeavour. For the undergraduate student considering undertaking a higher degree in a biological discipline, there are almost infinite choices of virus-based research projects to consider. Additionally, if one decides to study ancient symbiotic interactions between indigenous viruses and their hosts, there is a very good chance the researcher will have the opportunity to describe and understand one or more new-to-science species. We have only scratched the surface of the unique Australasian virome; the adage ‘more research is needed’ is especially true in the flora of this region.

This Special Issue provided a snapshot of some of the plant and fungal virological research being conducted in Australasia at the start of the third decade of the twenty-first century. Although much has been discovered, the field remains in its infancy. We are confident that future generations of virologists in the decades and centuries to come will build on what we have started, will endeavour to understand in greater detail the complex interactions between host and virus, between virus and virus, the evolutionary forces that act upon viruses when they jump hosts, as well as building better tools to detect and defeat the viruses that cause us harm.
